# Patient-Reported Outcomes of Orthodontic Treatment for Mild Crowding With Modified Aligner Appliance With Nickel-Titanium Springs: A Prospective Cohort Study

**DOI:** 10.7759/cureus.76179

**Published:** 2024-12-22

**Authors:** Ziad Mohamad Alhafi, Mohammad Y Hajeer, Ahmad S Burhan, Youssef Latifeh, Mowaffak A Ajaj, Alaa Oudah Ali Almusawi, Ahmad Salim Zakaria

**Affiliations:** 1 Department of Orthodontics, Faculty of Dentistry, University of Damascus, Damascus, SYR; 2 Department of Internal Medicine, Faculty of Medicine, University of Damascus, Damascus, SYR; 3 Department of Orthodontics, Faculty of Dentistry, University of Al-Knooz, Basrah, IRQ; 4 Department of Orthodontics, School of Dental Sciences, University Sains Malaysia, Kelantan, MYS

**Keywords:** chewing difficulty, discomfort, inman appliance, mild crowding, modified aligner appliance, nickle-titanium coil springs, pain, patient-reported outcomes, traditional orthodontic appliance, visual analog scale

## Abstract

Background and objectives

A modified aligner appliance with nickel-titanium springs (MAA) is a relatively new appliance that has not received extensive attention in orthodontics. This study evaluated the patient-reported outcomes when orthodontic treatment was provided using a modified aligner appliance to treat mild lower incisor crowding.

Materials and methods

This prospective cohort study consisted of 42 patients (11 males and 31 females; mean age 21.69 ± 2.56 years) with mild crowding. Twenty-one patients were treated using a modified aligner with nickel-titanium springs, while the remaining patients were treated with a conventional fixed orthodontic appliance (FA). Patient responses regarding pain, discomfort, and difficulty in chewing, swallowing, and speaking were recorded using a visual analog scale (VAS) at six assessment times: 24 hours (T1), two days (T2), three days (T3), two weeks (T4), one month (T5), and two months (T6) after appliance application.

Results

The mean perceived pain and discomfort levels were generally lower in the traditional fixed appliance (FA) group than the modified aligner appliance with nickel-titanium springs group at most assessment times. However, no statistically significant differences were found between the two groups. Chewing difficulty levels were similar between the two groups with no substantial differences. Swallowing and speech difficulty were significantly higher in the MAA group compared to the FA group during the first two days of treatment (P < 0.008).

Conclusions

Patients using both types of orthodontic appliances experienced temporary discomfort, including pain and difficulty chewing. While the modified aligners appliance with nickel-titanium springs caused additional challenges with swallowing and speaking, these issues were resolved over time, allowing patients to adjust to the treatment.

## Introduction

Pain is a highly personal experience that can vary widely from one person to another, even when caused by the same thing [1]. Factors such as age, gender, individual pain tolerance, emotional state, stress, the intensity of the stimulus, cultural background, and past pain experiences all contribute to how someone perceives pain [2,3]. While offering aesthetic and functional benefits, orthodontic treatment often has the downside of pain and discomfort [4-7]. Pain is a major obstacle to orthodontic treatment, often leading to decreased patient cooperation and treatment discontinuation [8-10]. Effective pain management is crucial for successful outcomes, as nearly all patients experience pain during treatment [3,11]. The pain patterns associated with traditional fixed appliances are well-documented [12,13], with peak discomfort occurring approximately 24 hours after treatment initiation and gradually subsiding [14]. While the initial pain of orthodontic treatment has been widely studied, a significant gap persists in our knowledge of how patients experience pain and discomfort as treatment progresses [3,15]. 

The choice of orthodontic treatment for a patient hinges on their specific diagnosis and the severity of their misaligned teeth [16]. Traditional fixed appliances remain the most common option, but their appearance and limited functionality can make them less appealing to patients [17]. Research has shown that different orthodontic treatments vary in cost, appearance, and treatment methods [18,19]. Orthodontics has made significant strides recently, with new techniques and materials constantly emerging [20]. While options like lingual and ceramic braces and clear aligners offer more discreet treatments, they often come with higher costs [20,21]. The spring aligner, a removable appliance used to correct mild crowding of anterior teeth, was introduced 25 years ago [22]. It was later modified by adding nickel-titanium springs to apply gentle and steady forces to the teeth [22,23]. However, despite its benefits, it may have some adverse effects on the surrounding soft tissues due to coiled springs, potentially promoting plaque accumulation and hindering effective oral hygiene [24].

Clinical trials have recently assessed the efficacy of a modified fixed version of this modified aligner appliance with nickel-titanium springs to treat mild crowding in the lower incisors. The findings demonstrated the effectiveness of this appliance in rectifying mild crowding in adults within a treatment timeframe comparable to that of traditional fixed orthodontic appliances [23,25]. A comparison of oral health-related quality of life (OHRQoL) between patients using fixed appliances and modified aligners with NiTi springs (MAA) revealed similar overall outcomes. However, patients with the MAA reported better psychological disability, while those with fixed appliances experienced fewer functional limitations [26]. The modified aligner with NiTi springs has become widely used in many countries' medical centers and teaching hospitals. While evaluating the effectiveness and feasibility of this technique is crucial, it is insufficient for determining its clinical applicability. Patient-centered outcomes, such as pain and discomfort associated with the method, should also be assessed. Therefore, the present study compared pain, discomfort, chewing, swallowing, and speech difficulties during treatment with the modified aligner appliance with NiTi springs and conventional fixed orthodontic appliances in a cohort of malocclusion patients with mild dental crowding.

## Materials and methods

Study design

This research was a prospective cohort study conducted at the Department of Orthodontics, University of Damascus, Syria. The University of Damascus's Local Research Ethics Committee approved the study (Approval number: UDDS-232519062022/SRC-223) on January 4, 2023. The University of Damascus provided financial support for the study (Grant number: 501100020595).

Sample size estimation

The sample size was estimated using Minitab® software (version 20.3, State College, PA, USA) based on a similar previous study [27]. Assuming a significance level of 5% and a study power of 95%, the minimum detectable difference in pain scores between the two groups was set at 2.5 cm on the visual analog scale (VAS). The standard deviation was 1.96 cm from the previous study [27]; the calculated sample size was 38 participants (19 per group). An additional two patients were added to each group to account for potential dropouts.

Study settings, participants, and inclusion criteria

Forty-two participants (11 males, 31 females) were included in this study, chosen from patients referred to the Orthodontics Department at Damascus University between September 2023 and April 2024. All the participants met the following inclusion criteria: adult patients aged 15 to 25, class I malocclusion with mild crowding of less than 4 mm, good oral hygiene, and no missing or extracted teeth (except for the third molars). The criteria for exclusion were patients with a bimaxillary protrusion, a history of previous orthodontic treatment, and severe skeletal discrepancy. All 42 patients received an information sheet about the objectives and methods of the study and provided written consent to participate. Patients in one of the two groups were included based on the patient's preference and the treating doctor's acceptance of this choice. Therefore, no randomization was applied when assigning patients to the two cohorts. Our orthodontic department accepts the two treatment protocols for this type of malocclusion; none is considered a "new" treatment.

The modified aligner appliance with NiTi springs group

Twenty-one patients were treated using a modified aligner appliance with NiTi springs (MAA). The MAA consisted of a vestibular acrylic pad and a lingual acrylic pad, which sit on the middle third of the labial and lingual surfaces of the anterior teeth. Closed-coil NiTi springs were embedded in the buccal wire segment, while open-coil NiTi springs were embedded in the lingual wire segment. The activation of these springs generated balanced and opposing forces on the surfaces of the anterior teeth, leading to the alignment of the teeth in the desired position. To fabricate the appliance, orthodontic bands were adapted onto the lower first permanent molars and an alginate impression was taken, ensuring the bands were captured in the impression. A silicone impression was taken to obtain a digital model. The orthodontic model was used with Blue Sky Plan software version 4.7 (Blue Sky Bio, Libertyville, IL, USA) to create a setup for tooth alignment and was then 3D printed in resin. The orthodontic bands were placed on this printed model, and the MAA appliance was fabricated.

**Figure 1:**
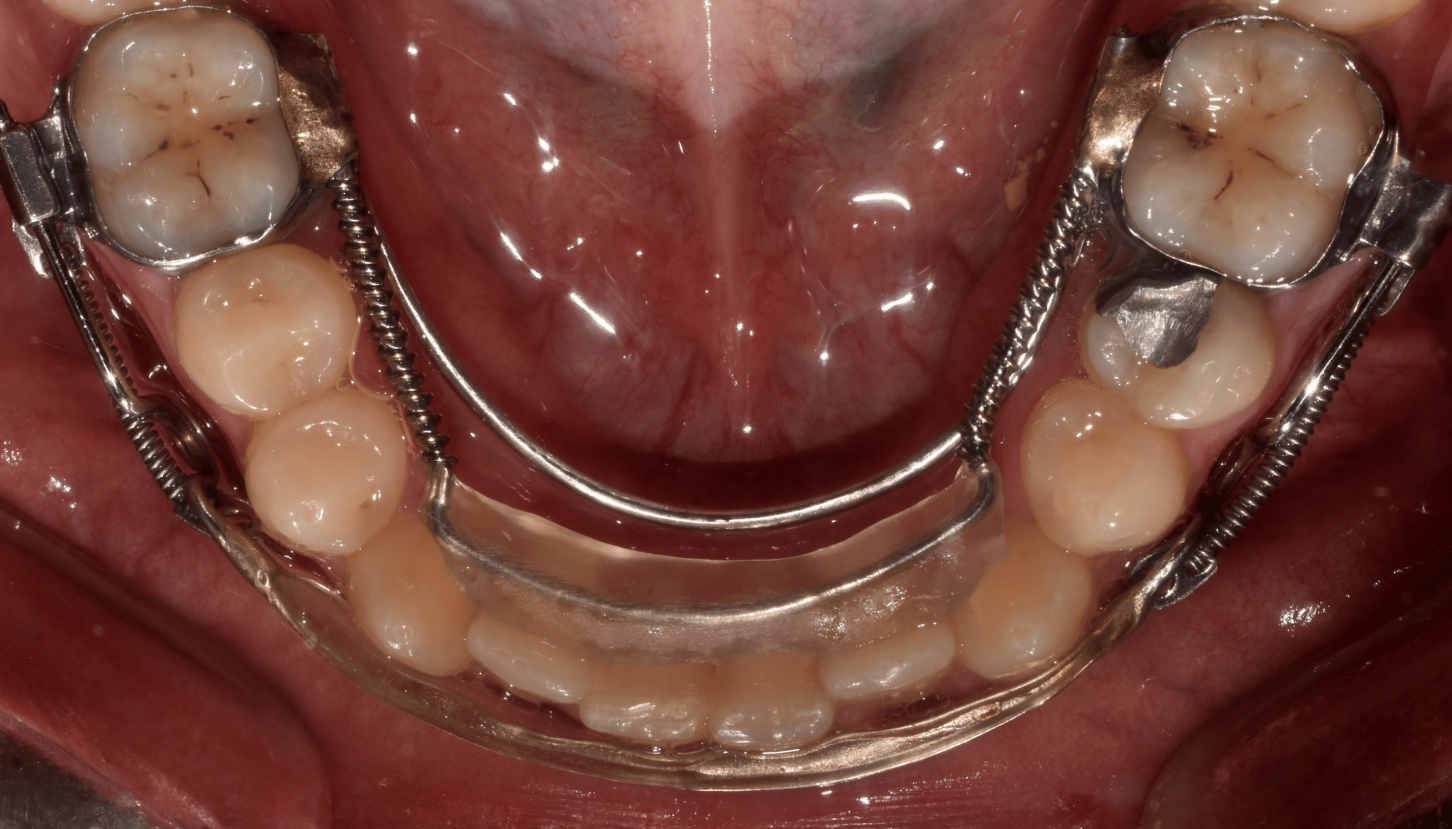
An intra-oral image of the modified aligner appliance with NiTi springs (MAA), which was used to align the lower anterior teeth

After intraoral appliance adjustment and verification of the absence of any complications like gingival or labial irritation, the appliance was removed to conduct interproximal reduction from canine to canine for securing the required space to align the teeth based on each patient's specific needs. This was achieved using double-sided metal abrasion strips. Subsequently, the bands were bonded with glass ionomer cement, and the appliance was placed (Figure 1). The amount of force applied by the NiTi springs was measured using an intraoral force gauge and adjusted to apply a force not exceeding 80 grams per side.

Traditional fixed appliances group

Twenty-one patients in this group received standard orthodontic treatment using brackets with a 0.022 x 0.028-inch slot and MBT prescription (Master Series®, American Orthodontics, Sheboygan, USA). The following sequence of orthodontic archwires was used: 0.012-inch Nickle-Titanium (NiTi), 0.014-inch NiTi, 0.016-inch NiTi, 0.016 x 0.022-inch NiTi, 0.016 x 0.022-inch stainless steel (SS), and then 0.017 x 0.025-inch SS wire (JISCOP, Gyeonggi-do, Korea). The archwire was replaced when the active wire approached or reached a state of neutrality. An interproximal reduction (IPR) was carried out following the same method used for the MAA group. In both groups, patients were monitored every two weeks throughout treatment to monitor treatment progress and make necessary adjustments.

Outcomes measures: the questionnaire

**Figure 2:**
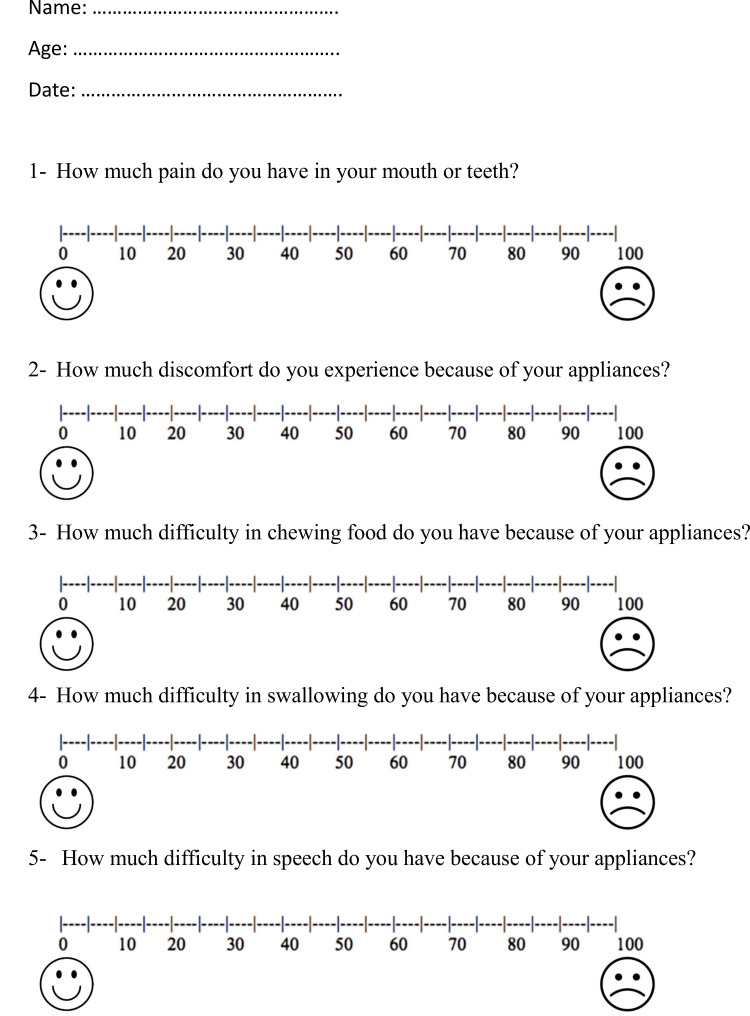
The questionnaire given to the patients in both groups at all assessment times.

The questionnaire was written in simple language that patients could easily understand. Patients filled out the questionnaire themselves while sitting in the dental chair. The researcher was available to answer questions but did not influence the patients' responses (Figure 2).

The questionnaire was given to patients at several time points: 24 hours (T1), two days (T2), and three days (T3) after the appliances were applied. It included five questions about pain, discomfort, chewing, swallowing, and speech difficulty. The same questionnaire was given again two weeks (T4), one month (T5), and two months (T6) after the appliances were applied.

A visual analog scale (VAS) was used to measure responses [11]. This is a 100-millimeter line with two ends: one for the lowest level and the other for the highest level of a particular feeling or sensation. For instance, to measure discomfort, point 0 represents no discomfort, and point 100 represents the worst possible discomfort. Participants marked a point on the line to indicate their current level of discomfort. The VAS score was obtained by measuring the distance, in millimeters, from the left anchor (representing no discomfort) to the point marked by the patient on the 100-mm visual analog scale.

Statistical analysis

SPSS® Version 26 program (IBM, Armonk, NY, USA) was used for the statistical analysis. The Shapiro-Wilk test was employed to assess the normality of data distribution. Mann-Whitney U tests were used to identify significant differences between the two groups. Friedman's test was used to determine if the variables had significant differences over time. Bonferroni's correction was applied to adjust the significance level for multiple pairwise comparisons. Results were considered statistically significant at a P ≤ 0.008.

## Results

Baseline sample characteristics

The study included 42 patients (11 males and 31 females), twenty-one patients in each group. The average age of the participants was 21.69 ± 2.56 years. All patients completed the study, and no one dropped out. Table 1 shows the patients' baseline demographics in the two cohort groups.

**Table 1:** Baseline sample characteristics MAA, modified aligner appliance with NiTi springs; FA, fixed appliances; SD, standard deviation; n, number of patients. † Employing chi-square test; ‡ employing independent sample t-test

Variable		MAA group (n=21)	FA group (n=21)	Sample (n= 42)	P-value
		n (%)	n (%)	n (%)	
Gender	Male	5 (23.8)	6 (28.6)	11 (26.2)	0.726^†^
	Female	16 (76.2)	15 (71.4)	31 (73.8)	
Age (years)	Mean ± SD	22.05 ± 2.47	21.33 ± 2.65	21.69 ± 2.56	0.373^‡^

Main findings

Pain Perception and the Levels of Discomfort

Both groups reported mild to moderate pain throughout the study. While the FA group generally experienced lower pain levels than the MAA group, these differences were not statistically significant (Table 2). Pain was highest on day two in both groups and decreased progressively over time (P <0.001; Table 2).

**Table 2:** Descriptive statistics of the levels of pain and discomfort at six assessment times in the two groups using visual analog scales and the p-values of significance tests. MAA: modified aligner appliance with NiTi springs; FA: fixed appliances; SD: standard deviation; T1: after 24 hours of the beginning of orthodontic treatment; T2: after 2 days; T3: after 3 days; T4: after 2 weeks; T5: after one month; T5: after two months. †Employing Friedman’s test (**significant at the 0.05 level); ‡ Employing Mann-Whitney U test (*significant at the 0.008 level).

Variable	Time point	MAA group (n=21)			FA group (n=21)			Mean difference	MAA vs. FA
		Mean ± SD	Median	P-value^†^	Mean ± SD	Median	P-value^†^		P-value^‡^
Pain	T1	53.33±13.16	50	<0.001**	47.14±11.01	50	<0.001**	6.19	0.105
	T2	58.10±14.01	60		55.95±10.67	50		2.14	0.583
	T3	38.57±16.51	40		37.62±8.30	40		0.952	0.663
	T4	20.95±11.36	20		23.81±8.64	30		-2.85	0.389
	T5	14.76±8.13	10		15.43±10.24	20		-0.66	0.752
	T6	11.43±11.95	10		9.86 ± 8.39	10		1.57	0.864
Discomfort	T1	63.81±13.59	60	<0.001**	58.10±10.77	60	<0.001**	5.71	0.179
	T2	69.43±12.28	70		66.67±13.54	70		2.76	0.492
	T3	50.48±15.64	50		49.52±16.87	50		0.95	0.681
	T4	28.43±13.14	30		24.29±12.87	20		4.14	0.267
	T5	16.67±12.38	20		13.29±10.18	10		3.38	0.297
	T6	10.95±7.68	10		8.10±9.28	10		2.85	0.155

Discomfort levels were moderate for the FA and MAA groups in the first three days. Subsequently, discomfort levels decreased gradually to mild levels during the remaining assessment times (P <0.001; Table 2). Mean discomfort levels were higher in the MAA group compared to the FA group at all follow-up times. However, no statistically significant differences were observed between the two groups (Table 2).

Chewing, Swallowing, and Speech Difficulties

In both the FA and MAA groups, mild to moderate difficulty in chewing was reported initially during the first two days. Subsequently, this difficulty decreased gradually throughout the remaining assessment times (P <0.001; Table 3). Mean chewing difficulty scores were lower in the FA group compared to the MAA group at all follow-up times. However, these differences were not statistically significant (Table 3).

**Table 3:** Descriptive statistics for chewing, swallowing, and speech difficulty levels at six assessment times in the two groups using visual analog scales and the p-values of significance tests. MAA: modified aligner appliance with NiTi springs; FA: fixed appliances; SD: standard deviation; T1: after 24 hours of the beginning of orthodontic treatment; T2: after 2 days; T3: after 3 days; T4: after 2 weeks; T5: after one month; T5: after two months. †Employing Friedman’s test (**significant at the 0.05 level); ‡ Employing Mann-Whitney U test (*significant at the 0.008 level).

Variable	Time point	MAA group (n=21)			FA group (n=21)			Mean difference	MAA vs. FA
		Mean ± SD	Median	P-value^†^	Mean ± SD	Median	P-value^†^		P-value^‡^
Chewing difficulty	T1	52.86±15.53	50	<0.001**	50.48±8.64	50	<0.001**	2.38	0.835
	T2	56.43±14.41	50		52.38±9.43	50		4.04	0.444
	T3	38.57±16.21	40		38.10±9.80	40		0.47	0.676
	T4	20.48±10.71	20		19.52±14.31	20		0.95	0.786
	T5	11.43±9.63	10		12.14±9.29	20		-0.71	0.766
	T6	6.19±7.40	0		5.24±7.49	0		0.95	0.610
Swallowing difficulty	T1	55.71±13.25	50	<0.001**	42.86±11.89	40	<0.001**	12.85	0.004*
	T2	51.43±19.56	50		32.86±12.70	30		18.57	0.001*
	T3	30±20.97	30		23.33±16.22	30		6.66	0.311
	T4	14.05±12.80	10		9.52±7.40	10		4.52	0.263
	T5	10±10	10		5.71±6.76	0		4.28	0.175
	T6	6.67±7.30	10		4.29±5.07	0		2.38	0.329
Speech difficulty	T1	64.29±17.48	60	<0.001**	41.90±12.09	40	<0.001**	22.38	<0.001*
	T2	61.43±22.20	60		34.76±12.09	40		26.66	<0.001*
	T3	40±21.90	30		24.76±16.31	20		15.23	0.020
	T4	21.19±15.64	20		15.71±12.47	10		5.47	0.231
	T5	12.38±10.91	10		6.19±8.04	0		6.19	0.057
	T6	7.62±8.89	0		3.33±5.77	0		4.28	0.110

Patients in the MAA group reported moderate difficulty in swallowing during the first two days. This difficulty decreased significantly to mild levels by the end of the follow-up period (P <0.001; Table 3). Overall, mean swallowing difficulty scores were significantly greater in the MAA group compared to the FA group on the first and second days after appliance application (P = 0.004 and P = 0.001, respectively; Table 3). The MAA group experienced moderate initial speech difficulty, which decreased significantly over time (P <0.001; Table 3). Compared to the FA group, the MAA group had significantly higher speech difficulty scores on the first two days of treatment (P <0.001).

## Discussion

This study evaluated patient-reported outcome measures (PROMs) associated with using a modified aligner with nickel-titanium springs and conventional fixed orthodontic appliances. Optimal orthodontic results rely on techniques and appliances that minimize patient discomfort and pain. Pain is a common problem that may affect patient cooperation and lead to adverse treatment outcomes, so it is crucial to address this issue [11]. The modified aligner appliance with nickel-titanium spring was chosen for the current study due to its proven efficacy as an aesthetic treatment modality for mild crowding cases [23,25].

In both groups, peak pain levels were observed on the second day, with a significant increase in pain intensity from day one, reaching a maximum on the second day. Subsequently, pain levels began to decrease, reaching their lowest point two months after the application of the orthodontic appliances. This finding is consistent with previous studies that evaluated pain levels with conventional fixed orthodontic appliances, which found that pain peaks one day after applying orthodontic forces and then gradually decreases [28,29].

Discomfort is the patient's feeling of unease at rest, unrelated to the basic functions of the oral cavity, such as swallowing, chewing, or speaking. It was observed that discomfort peaked on the second day in both groups and then began to decrease with statistically significant differences gradually. This can be explained by the occurrence of adaptation and habituation that is commonly observed after a period of orthodontic appliance application. This reduces the patients' discomfort after a month of treatment initiation [30]. The moderate levels of chewing difficulty immediately after MAA application and on the second day can be attributed to the anatomical proximity of the lingual aspect of the appliance to the active elements of mastication, namely the tongue, and teeth. However, the rapid decrease in discomfort levels after that is likely due to adaptation and habituation to the appliance's presence [31,32].

Swallowing difficulty levels in the MAA group were significantly higher than the FA group on both the first and second days. No significant differences were observed at other assessment time points. The observed differences between the two groups immediately after appliance placement and the following day can be attributed to the MAA appliance containing a lingual component that may hinder tongue movement before the patient becomes accustomed to the appliance [33].

The study results demonstrated statistically significant differences between the two groups regarding speech difficulty during the initial assessment periods (day one and day two). Specifically, the MAA group exhibited significantly higher difficulty levels in speech articulation than the FA group. However, no significant differences were observed at subsequent assessment time points. The observed differences between the two groups immediately following appliance placement can be attributed to an acrylic pad between the buccal surfaces of the lower incisors and the lower lip, which may initially hinder the articulation of certain sounds. Patients typically require an adjustment period to accommodate this new appliance [26,34].

Limitations of the current study

This is the first study to investigate the pain, discomfort, and functional issues associated with the MAA appliance, but it has some limitations. One limitation of the present study was the lack of subgroup analysis to differentiate between male and female responses to the appliance. Furthermore, the patients' responses to the questionnaire at different time points were used to evaluate the outcomes. Several factors may have influenced their perceptions at the different activation points in the study. Finally, this study focused only on patient-reported outcomes. Therefore, future studies must assess other variables, such as gingival and periodontal status, after using these appliances.

## Conclusions

Both the modified aligner appliance and the traditional fixed orthodontic appliance were associated with mild to moderate pain, discomfort, and difficulty chewing but without any adverse effects. The modified aligner appliance with nickel-titanium springs caused moderate difficulty swallowing and speaking during the first two days, but this gradually decreased during follow-up periods.
